# Mobile Peer-Support for Opioid Use Disorders: Refinement of an Innovative Machine Learning Tool

**DOI:** 10.20900/jpbs.20200001

**Published:** 2020-02-03

**Authors:** Caroline R. Scherzer, Megan L. Ranney, Shrenik Jain, Satya Prateek Bommaraju, John Patena, Kirsten Langdon, Evelyn Nimaja, Ernestine Jennings, Francesca L. Beaudoin

**Affiliations:** 1 Department of Emergency Medicine, Rhode Island Hospital, 593 Eddy Street, Providence, RI 02903, USA; 2 Department of Psychiatry, Rhode Island Hospital, 593 Eddy Street, Providence, RI 02903, USA; 3 Department of Emergency Medicine, Alpert Medical School of Brown University, 55 Claverick Street 2nd Floor, Providence, RI 02903, USA; 4 Marigold Health, 2 Ave de Lafayette, Boston, MA 02111, USA; 5 Department of Psychiatry and Human Behavior, Alpert Medical School of Brown University, 700 Butler Drive, Providence, RI 02906, USA; 6 Center for Behavioral and Preventive Medicine, The Miriam Hospital, Alpert Medical School of Brown University, 167 Point Street, Providence, RI 02903, USA; 7 Department of Health Services, Policy, and Practice, Brown School of Public Health, 121 South Main Street, Providence, RI 02912, USA

**Keywords:** opioid use disorder, peer support, machine-learning, technology, natural language processing, mobile treatment, application

## Abstract

**Background:**

The majority of individuals with Opioid Use Disorder (OUD) do not receive any formal substance use treatment. Due to limited engagement and access to traditional treatment, there is increasing evidence that patients with OUDs turn to online social platforms to access peer support and obtain health-related information about addiction and recovery. Interacting with peers before and during recovery is a key component of many evidence-based addiction recovery programs, and may improve self-efficacy and treatment engagement as well as reduce relapse. Commonly-used online social platforms are limited in utility and scalability as an adjunct to addiction treatment; lack effective content moderation (e.g., misinformed advice, maliciousness or “trolling”); and lack common security and ethical safeguards inherent to clinical care.

**Methods:**

This present study will develop a novel, artificial-intelligence (AI) enabled, mobile treatment delivery method that fulfills the need for a robust, secure, technology-based peer support platform to support patients with OUD. Forty adults receiving outpatient buprenorphine treatment for OUD will be asked to pilot a smartphone-based mobile peer support application, the “Marigold App”, for a duration of six weeks. The program will use (1) a prospective cohort study to obtain text message content and feasibility metrics, and (2) qualitative interviews to evaluate usability and acceptability of the mobile platform.

**Anticipated findings and future directions:**

The Marigold mobile platform will allow patients to access a tailored chat support group 24/7 as a complement to different forms of clinical OUD treatment. Marigold can keep groups safe and constructive by augmenting chats with AI tools capable of understanding the emotional sentiment in messages, automatically “flagging” critical or clinically relevant content. This project will demonstrate the robustness of these AI tools by adapting them to catch OUD-specific “flags” in peer messages while also examining the adoptability of the platform itself within OUD patients.

## INTRODUCTION

Adjuncts to treatment for opioid use disorder (OUD) are urgently needed. Opioid overdoses are a leading cause of death for Americans under 50 years old, with recent years recording the most opioid overdose deaths on record [1,2]. The opioid epidemic has not abated despite a recent overall decrease in the number of opioid analgesics prescribed by US providers [3–6]. Heroin use is increasing for the first time in more than a decade [7–13], and overdose deaths due to potent synthetic opioids, such as fentanyl and carfentanil, are on the rise [14–20]. In light of this public health crisis, there is a pressing need for novel approaches to treat patients with OUDs.

Peer support programs may increase efficacy of and retention in OUD treatment. Peer-based interventions have been shown to be an effective component of care provision across multiple health settings and conditions, including addiction [21–26]. There is strong evidence to support the role of peer-delivered behavioral interventions for OUD in both clinical and non-clinical settings (e.g., narcotics anonymous) [9].

Novel delivery can improve access to peer support. Technology is already used to augment addiction treatment programs, ranging from automated SMS reminders to self-guided online treatments [27–31]. However, these “self-help” mechanisms do not offer the same advantage of having two-way, peer-to-peer to communication. Text-based peer support can be used to drive engagement and retention in structured treatment programs [28]. Text communications between peers can be done using a pseudonym (with only the moderator or provider being aware of the patient’s identity), thereby increasing patients’ comfort. The process of typing and rereading one’s messages may also improve awareness, akin to therapeutic “homework” such as journaling. Additionally, vulnerable populations, including low-income and low-literacy patients, have demonstrated a preference for the text-message medium [32,33]. Millions of individuals already use ad-hoc online forums (e.g., Reddit™) to obtain peer support and health information indicating the demand for this type of platform. Unfortunately, existing sites are plagued by intentionally malicious or inappropriate users (“Trolls”) and lack connection to any larger clinical infrastructure or oversight. Current text-based psychotherapy apps such as Talkspace and BetterHelp only offer individual therapy at a high cost (~$200/month), that is economically limiting for many patients [34,35].

Artificial Intelligence (AI) can offer continuous, real-time access to monitored peer-support in a platform that is scalable and economical. AI involves analyzing large amount of data with algorithms that automatically adjust, or “learn”, as they are exposed to new information, drawing inferences unclear to a standard analysis. Natural language processing (NLP) is the subset of AI focusing on human language [36,37]. A body of previous work has established a computational link between language patterns and the emotional sentiment behind the language (e.g., self-harm, malicious intent) [36,38–40]. Correctly classifying the intended sentiment of a text message allows the application to “flag” specific content. The use of NLP on messages within the peer support groups may provide significant benefits to users by proactively detecting when they might need immediate provider attention and ensuring peer-peer interactions are appropriate.

We propose a novel, machine-learning enabled, mobile treatment delivery method that fulfills the need for a robust, secure, technology-based peer support platform. The present “Marigold App” is an online peer-support platform that offers layers of innovation that are not readily available in other treatment programs or online forums: (1) continuous, economical, real-time access to supervised peer-support, (2) a monitored, high fidelity mobile environment with built in “flags” for specific risky behaviors, and (3) scalability. We plan to refine the existing application, currently used for mental health, to enable its use as an adjunct to treatment for OUD. This technology would allow outpatient treatment centers to overcome many patient-level barriers to retaining patients in OUD treatment (e.g., transportation, stigma) while also overcoming provider-level barriers (lack of workforce for real-time 24/7 monitoring of online conversations). We will develop a mobile application that allows patients to reach out for care at any time, from anywhere, with reduced concerns for stigma. The platform also can offer support during high-risk periods that occur outside of structured treatment settings. High risk periods may include the experience of cravings or urges to use substances. By enabling dynamic, continuous assessment of patient relapse risk, and by automatically notifying providers when a patient needs more intensive care resources, it will provide a safe and accessible format for peer support. Finally, the Marigold App has the potential for customization, including: (a) giving providers access to content discussed in peer support groups, improving the ability to address patient-specific concerns and needs; and, (b) passively tracking patient progress over time and adapting the algorithms to work on peer-group specific jargon. This platform is uniquely scalable due to the NLP technology. It will be the first of its kind to model language nuances specific to OUD and relapse. These machine-learning algorithms can provide automation and scale that could not be matched by humans monitoring 24/7 text conversations. This will allow existing peer recovery specialists to support larger patient capacities.

### Specific Aims

There are two specific aims of this project. First, we aim to develop and optimize NLP algorithms to detect text messaging content that may signal relapse or impending relapse in patients in recovery from OUDs. The goal of the flagged content is to have the NLP-based algorithms approximate what a human (and specifically a clinician) might consider concerning text message sentiment. For instance, if a user messages “I feel like I can’t do this anymore”, the NLP-based algorithm should be both sensitive and specific to the sentiment in the text, not the outcome per say (e.g., at high suicide risk). Outcomes assessment (e.g., suicide risk) would still rely on a clinician assessment. Future work could evaluate and validate whether text message sentiment can accurately predict adverse mental health outcomes such as suicide risk or relapse. In future work, we will attempt to uncover correlations between language and specific sentiment in: (1) recurrent use of opioids or other substances (2) craving or urge to use opioids (3) pain (4) negative affect. The first domains are two explicit signals of relapse or impending relapse, whereas perception of poorly controlled pain and negative affect are predictive of relapse. Message data generated by the participants will be anonymized, tagged, and categorized by speech analysts to provide training data to identify flags specific to OUD. The NLP models will then be tested in our existing database of 100,000 text messages. On average, 1–2 messages per participant per day will need to be provided by each participant to yield effective AI procedures. Second, we aim to demonstrate feasibility and acceptability of Marigold Health’s mobile peer support app as an adjunct to existing OUD treatment. In accordance with our prior work, primary outcomes will be feasibility and acceptability. We will also interview users of the app to explore qualitative satisfaction and to obtain feedback on user experience and feature refinement, using Marigold Health’s and the Co-Is’ standard qualitative assessment protocols.

## METHODS

### Study Setting and Patient Population

Participants will be recruited to enroll in the Marigold App at Rhode Island Hospital’s Lifespan Recovery Center, a hospital-affiliated outpatient clinic that offers medications for OUD (OUD; e.g., buprenorphine) and ancillary supports for patients with OUDs. In Rhode Island, the prevalence of OUDs and incidence of opioid overdoses are some of the highest in the country [41]. Similar studies have had high rates of recruitment and retention (>90% retention in studies of text-message-based behavioral health interventions) [42–46]. The average number of new patient admissions at the Lifespan Recovery Center is ~30 per month, and there is ample opportunity to recruit participants during treatment. This will offer the opportunity to observe interactions between individuals maintained in treatment and those who recently initiated treatment. Such dynamics open the window to capture more insights. Additionally, the center has established a research protocol within the clinic to evaluate patient- and program-level outcomes: to date, 92% of patients approached have agreed to participate in research-related activities. Participant characteristics include: 40% female; mean age 44 (range 20–76); ethnic and racial distribution as follows, 20% Hispanic, 84% White, 7% Black/African American, 7% more than one race, 1% Native Hawaiian/Pacific Islander, and 1% American Indian/Alaskan Native.

### Preliminary Work

**Figure 1.**
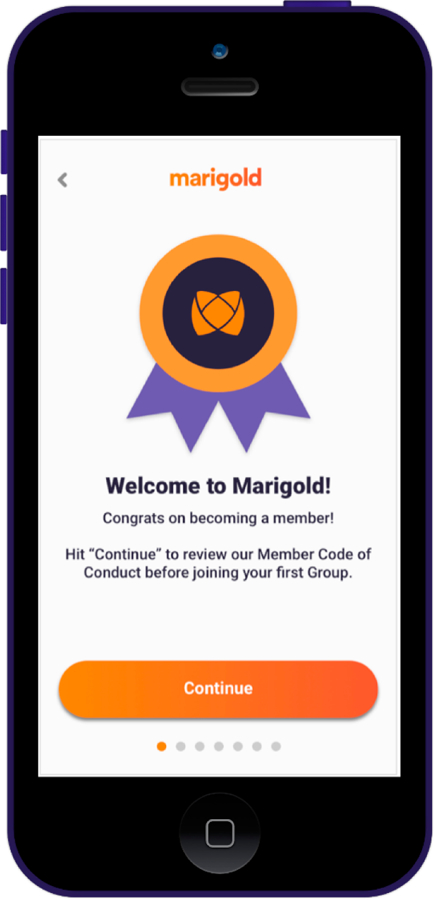
User Screen.

**Figure 2.**
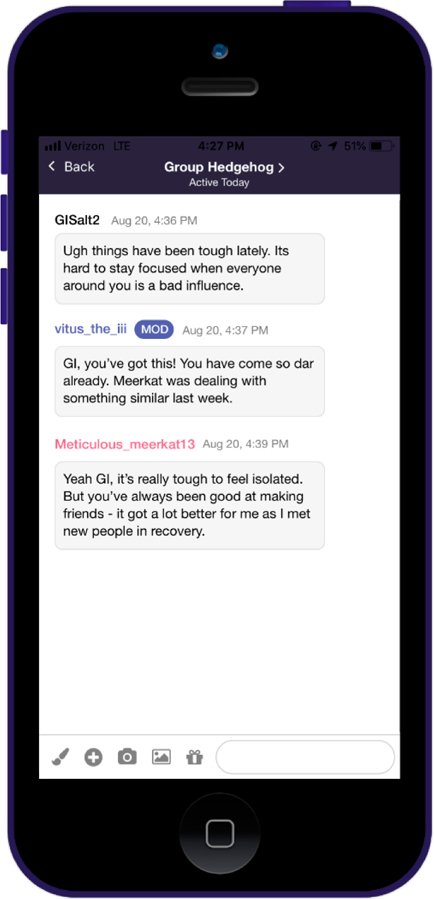
Moderator Screen.

Marigold Health’s current mobile app (see Figure 1) allows text-based group therapy and peer support for individuals with depression and anxiety. The Marigold platform is ultimately intended for integration within the workflow and electronic health system of a care management team within a clinic or health system. The Marigold App can also integrate clinician-selected handouts, diagnostic forms, and polls, to facilitate the easy assessment of users in-app. In the application, users choose a support group after enrollment; this is typically based on characteristics of the group or after a one-on-one in-app chat with the group’s moderator. Each support group consists of 5–9 users and a trained peer-support specialist. Patients’ personal information is only visible to group moderators; peer users only see a user-chosen, pseudonym. App moderators facilitate the flow of text-conversations in-app (see Figure 2), and can text single or multiple group members.

**Figure 3.**
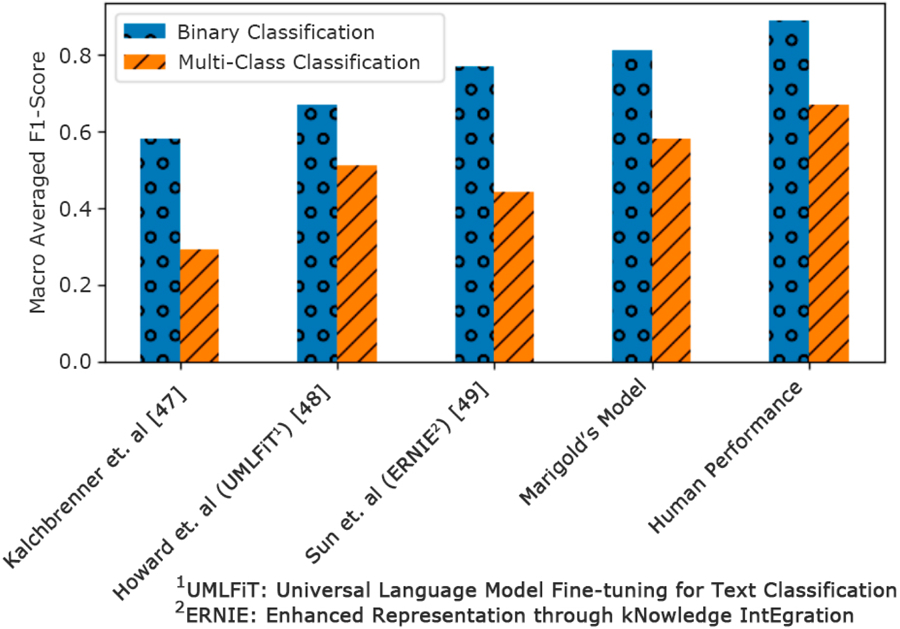
Performance of Marigold’s Machine Learning Model versus Other Published Models and Human Performance.

The current version of the app is live at a dozen sites (~1000 users) demonstrating stability and reliability of the software. Marigold Health’s NLP analytics are already highly accurate at detecting messages that require immediate provider intervention. NLP algorithms identify two “red flag” types of content within peer chats: (1) expressed or implied intent to harm self or others (2) malicious conduct or “trolling”. To determine when content requires clinical oversight, NLP engineers correlations between language and specific sentiment by sorting sentences into binary (needs intervention aka “flag” or not) or multiple (level of severity) classification. Figure 3 depicts the performance of Marigold’s Models on two classification tasks (binary and multi-class) when compared to state-of-the-art research methods. In the binary case the model is predicting whether a given message needs moderator intervention or not. Moderator intervention occurs in cases of self-harm, harm to others, risk of relapse, etc. For the multi-class problem, the model also predicts the severity of the message on a scale from 1 to 5. The metric used for evaluation is the Macro Averaged F1 score. The F1 score is computed as the weighted average of precision and recall and considers both false positives and false negatives into account. This F1 score is computed for each class individually and then simply averaged to compute the final Macro Averaged F1 Score. We also show the average performance of human annotators when attempting the same task.

### Intervention Procedures

Recruitment will occur in two ways: (1) a prospective cohort study to obtain text message content and feasibility metrics and (2) qualitative interviews to evaluate usability and acceptability of the app itself. Participant recruitment will occur during the course of usual care at the Lifespan Recovery Center. The Marigold app will serve as an adjunct to usual care, which may include medication and additional services such as counseling, case management, and peer support. Participants will be eligible for enrollment if they are an English-speaking, adult (≥18 years-old) and meet DSM-5 diagnostic criteria for OUD per their treating provider at the Lifespan Recovery Center. The app is not currently available for non-English speakers, but other languages will be an area of future development. Participants will be excluded from the study if they do not have an Android or iOS platform smartphone, are pregnant, incarcerated, or unable to provide informed consent.

The study research assistant will recruit directly from the population of patients actively engaged in OUD treatment at the Lifespan Recovery Center. All eligible patients will be approached to participate in the program. After obtaining written and informed consent, participants will be administered a baseline assessment. The baseline assessment will be conducted on touchscreen tablets using REDCap (a HIPAA compliant online data collection system) [50] that allows for direct, secure, and remote data entry. Assessments will include measures of: socio-demographic variables; medical history (including history of chronic pain and mental health), history of substance use, and treatment history (e.g., type and duration of treatment). Upon completion of the baseline assessment, the Marigold App will be downloaded onto the participant’s smartphone. Participants will be placed in peer support groups of 5–9 based on chronological order and on a rolling basis. Groups will be moderated by the study coordinator and research assistant. The research assistant will regulate the flow of conversation with standard text language developed by the study team, monitor for use, and respond to any generated “flags” according to the human subject’s safety protocol. The small business has the infrastructure to monitor for flagged content 24/7 and the investigative team will utilize an on-call system to respond in real time. All text messages (non-flagged content) will also be reviewed by the study team within 24 h.

Participants must maintain a minimum level of weekly in-app activity (an average of >1 message/day) to be considered “active”. Participants will be compensated for their time $40 for the initial enrollment and then $5 daily for each day that the app is actively used, up to $250 in total. Moderators will attempt to engage inactive users, though users will have their accounts deactivated after two weeks of inactivity. In the event of ‘trolling’ the moderators will receive a flag and can respond in real-time to individual users or can escalate a behavioral health crisis to a local crisis team. Content flagged for suicidal or homicidal ideation is sent to a clinician for review in real time. Other content (e.g., trolling) is sent to the moderator in real time.

We will collect data on feasibility: study recruitment and refusal rates, program completion, follow-up rates, number and length of messages generated, and rates of study attrition. Primary outcomes will be feasibility (75% consenting; 80% retained at 4 weeks) and acceptability (mean of 75% logging in daily; system usability scores >80%, high qualitative satisfaction). At the end of the 6-week study period, participants will be asked to complete a web-based survey to measure participant acceptance as assessed by (1) System Usability Scale, a participant-completed, validated metric for measuring technologies’ usability and acceptability [51,52], in which higher scores equate to higher usability [53,54]; (2) A modified version of the Client Satisfaction Questionnaire-8 [55], a validated measure of intervention satisfaction previously used by our team. If participants do not complete the web-based survey, they will be contacted by phone.

At the close of the six-week period, we will conduct qualitative interviews to refine the user interface of the application. Participants will be recruited from the Lifespan Recovery Center, as described above. Recruitment will occur purposefully, to ensure equal numbers of each sex and to represent patients in a variety of stages of change (e.g., new to treatment, in long-term treatment) and will continue until thematic saturation is reached. We will first test features of the app and document common user actions in-app, with each successive qualitative interview reviewing technical changes implemented since the previous one. We will follow the Think Aloud™ protocol for evaluating technical interfaces, in which individuals first verbalize their thoughts as they navigate the platform. Following this, participants will be interviewed and asked what they thought was missing or any difficulties they had. We will also show patients samples of engaging text messages, and note their responses (e.g., encouraging, overbearing) before adjusting messages for the next group. Participants will be compensation $40 for their participation in qualitative interviews.

## DATA ANALYSIS

### Quantitative Assessment of Feasibility and Acceptability

Message data generated on the platform will be anonymized and tagged to (1) provide OUD-specific data to train existing models on trolling, suicidal or homicidal ideation, and (2) engineer new features as correlations between language and specific sentiment is uncovered, specifically: (1) recurrent use of opioids or other substances, (2) craving or urge to use opioids, (3) pain, and (4) negative affect. The first two domains are explicit signals of relapse or impending relapse, whereas perception of poorly controlled pain and negative affect are predictive of relapse [56,57]. We will split tagged data into a training set, to improve our algorithm on, and a testing set, to evaluate our M-F1 score on. As we train, we will also develop new features (e.g., “shaking or sweating are symptoms of withdrawal”). The larger data corpus obtained from this specific aim will allow us to test novel features that encode context and state of the user’s drug habits. Examples of these features would be techniques to identify slang or euphemisms referring to drugs (“I want some brown sugar”) or identifying whether users are at risky locations (if a user at risk of suicide is going to the roof of a building). Coding and analysis will be completed by the Marigold Health team.

### Qualitative Assessment of Feasibility and Acceptability

Analysis will result in aggregated preferences and recommendations about message components. Interviews will be digitally recorded and transcribed. Using the technique of thematic analysis, categories and sub-categories related to the outcomes of interest will be grouped, or “coded” [58,59], deductive codes will be drawn from the interview guide topics (e.g., participant understanding of intervention design, message content and purpose), and inductive codes will capture additional themes that emerge from the participants. All transcripts will be independently double coded and then compared to ensure comprehensiveness. Agreed-upon codes will be entered into NVivo qualitative software [60]. Thematic summaries, describing the range of data in each code, will be discussed among the entire team, and used to adapt and refine the intervention. An audit trail of coding decisions and other aspects of analysis will be kept.

## DISCUSSION

The Marigold App will allow patients to access a tailored support group 24/7, and is augmented with AI tools capable of understanding the emotional sentiment in messages, automatically “flagging” critical or clinically relevant content, creating a scalable system to keep groups safe and constructive. This project plans to demonstrate the robustness of these AI tools by adapting them to catch OUD-specific “flags” in peer messages while also examining the adoptability of the platform itself within OUD patients. This novel machine learning solution is poised to increase accessibility to treatment for OUD, the fastest-rising source of American morbidity and mortality. As healthcare systems and payers assume more risk for OUD patients, there is also a strong commercial potential for a mobile platform that can rapidly, ethically, and effectively deliver a moderated peer support community as an adjunct to standard treatment for OUD. One potential downside to moderation is that some users may be less inclined to use the app. The Marigold App offers continuous, economical, real-time access to supervised peer-support and has the potential to improve treatment outcomes, which in turn, could reduce future incidence of overdose and death.

A future proposal will conduct a fully-powered RCT to determine this technology’s effect on patient outcomes (retention in OUD treatment and relapse); to quantify potential cost savings; and to further enhance our nuanced NLP tools capable of normalizing to specific individuals, demographic factors, and populations.

## References

[1] Overdose Death Rates. 2017 Available from: https://www.drugabuse.gov/related-topics/trends-statistics/overdose-death-rates Accessed 2017 Jan 7.

[2] Rudd RA, Aleshire N, Zibbell JE, Gladden RM. Increases in Drug and Opioid Overdose Deaths--United States, 2000–2014. Morb Mortal Wkly Rep. 2016;64:1378–82. 10.15585/mmwr.mm6450a3 26720857

[3] Wu LT, Zhu H, Swartz MS. Treatment utilization among persons with opioid use disorder in the United States. Drug Alcohol Depend. 2016;169:117–27. 27810654 10.1016/j.drugalcdep.2016.10.015 PMC5223737

[4] IMS Institute for Healthcare Informatics. Medicines Use and Spending in the U.S.A Review of 2015 and Outlook to 2020. City (Country): IMS Institute for Healthcare Informatics; 4 2016 Available from: http://www.imshealth.com/en/thought-leadership/quintilesims-institute/reports/medicines-use-and-spending-in-the-us-a-review-of-2015-and-outlook-to-2020 Accessed 2016 Dec 6.

[5] Kertesz SG. Turning the Tide or Riptide? The Changing Opioid Epidemic. Subst Abus. 2017;38(1):3–8. doi: 10.1080/08897077.2016.1261070 27858590

[6] Hirsch A, Proescholdbell SK, Bronson W, Dasgupta N. Prescription histories and dose strengths associated with overdose deaths. Pain Med. 2014;15:1187–95. 25202775 10.1111/pme.12391

[7] Rudd RA, Seth P, David F, Scholl L. Increases in Drug and Opioid-Involved Overdose Deaths—United States, 2010–2015. Morb Mortal Wkly Rep. 2016;65:1445–52. 10.15585/mmwr.mm655051e1 28033313

[8] Vaughn MG, Salas-Wright CP, Oh S. Trends in heroin access among adolescents in the United States, 2002–2014. Prev Med. 2017;99:67–8. 28189804 10.1016/j.ypmed.2017.01.019

[9] Meiman J, Tomasallo C, Paulozzi L. Trends and characteristics of heroin overdoses in Wisconsin, 2003–2012. Drug Alcohol Depend. 2015;152:177–84. 25935735 10.1016/j.drugalcdep.2015.04.002

[10] Jones CM, Logan J, Gladden RM, Bohm MK. Vital Signs: Demographic and Substance Use Trends Among Heroin Users—United States, 2002–2013. Morb Mortal Wkly Rep. 2015;64:719–25. PMC4584844 26158353

[11] Lipari RN, Hughes A. Trends in Heroin Use in the United States: 2002 to 2013 CBHSQ Report. Rockville (MD, US): The Substance Abuse and Mental Health Services Administration; 2013. 26913325

[12] Barrio G, Montanari L, Bravo MJ, Guarita B, de la Fuente L, Pulido J, Trends of heroin use and heroin injection epidemics in Europe: findings from the EMCDDA treatment demand indicator (TDI). J Subst Abuse Treat. 2013;45(1):19–30. doi: 10.1016/j.jsat.2012.11.002 23462151

[13] Victor GA, Walker R, Cole J, Logan TK. Opioid analgesics and heroin: Examining drug misuse trends among a sample of drug treatment clients in Kentucky. Int J Drug Policy. 2017;46:1–6. 28511053 10.1016/j.drugpo.2017.01.008

[14] Swanson DM, Hair LS, Strauch Rivers SR, Smyth BC, Brogan SC, Ventoso AD, Fatalities Involving Carfentanil and Furanyl Fentanyl: Two Case Reports. J Anal Toxicol. 2017;41(6):498–502. doi: 10.1093/jat/bkx037 28575422

[15] Dwyer JB, Janssen J, Luckasevic TM, Williams KE. Report of Increasing Overdose Deaths that include Acetyl Fentanyl in Multiple Counties of the Southwestern Region of the Commonwealth of Pennsylvania in 2015–2016. J Forensic Sci. 2018;63(1):195–200. doi: 10.1111/1556-4029.13517 28605020

[16] Marshall BDL, Krieger MS, Yedinak JL, Ogera P, Banerjee P, Alexander-Scott NE, Epidemiology of fentanyl-involved drug overdose deaths: A geospatial retrospective study in Rhode Island, USA. Int J Drug Policy. 2017;46:130–5. doi: 10.1016/j.drugpo.2017.05.029 28601512

[17] Carroll JJ, Marshall BDL, Rich JD, Green TC. Exposure to fentanyl-contaminated heroin and overdose risk among illicit opioid users in Rhode Island: A mixed methods study. Int J Drug Policy. 2017;46:136–45. doi: 10.1016/j.drugpo.2017.05.023 28578864 PMC5560423

[18] Somerville NJ, O’Donnell J, Gladden RM, Zibbell JE, Green TC, Younkin M, Characteristics of Fentanyl Overdose—Massachusetts, 2014–2016. Morb Mortal Wkly Rep. 2017;66:382–6. 10.15585/mmwr.mm6614a2 PMC5657806 28406883

[19] Mercado MC, Sumner SA, Spelke MB, Bohm MK, Sugerman DE, Stanley C. Increase in Drug Overdose Deaths Involving Fentanyl-Rhode Island, January 2012–March 2014. Pain Med. 2018;19(3):511–23. doi: 10.1093/pm/pnx015 28340233 PMC5587352

[20] Bode AD, Singh M, Andrews J, Kapur GB, Baez AA. Fentanyl laced heroin and its contribution to a spike in heroin overdose in Miami-Dade County. Am J Emerg Med. 2017;35(9):1364–5. doi: 10.1016/j.ajem.2017.02.043 28268113

[21] Boisvert R, Martin L, Grosek M, Clarie A. Effectiveness of a peer-support community in addiction recovery: participation as intervention. Occup Ther Int. 2008;15:205–20. 18844242 10.1002/oti.257

[22] Deering K, Kerr T, Tyndall M, Montaner JS, Gibson K, Irons L, A peer-led mobile outreach program and increased utilization of detoxification and residential drug treatment among female sex workers who use drugs in a Canadian setting. Drug Alcohol Depend. 2011;113:46–54. 20727683 10.1016/j.drugalcdep.2010.07.007

[23] Treloar C, Rance J, Bath N, Everingham H, Micallef M, Day C, Evaluation of two community-controlled peer support services for assessment and treatment of hepatitis C virus infection in opioid substitution treatment clinics: The ETHOS study, Australia. Int J Drug Policy. 2015;26:992–8. 25697089 10.1016/j.drugpo.2015.01.005

[24] Bassuk EL, Hanson J, Greene RN, Richard M, Laudet A. Peer-Delivered Recovery Support Services for Addictions in the United States: A Systematic Review. J Subst Abuse Treat. 2016;63:1–9. 26882891 10.1016/j.jsat.2016.01.003

[25] James T, Bibi S, Langlois B, Dugan E, Mitchell P. Boston Violence Intervention Advocacy Program: A Qualitative Study of Client Experiences and Perceived Effect. Acad Emerg Med. 2014;21:742–51. 25039818 10.1111/acem.12409

[26] Gonzalez SA, Fierer DS, Talal AH. Medical and Behavioral Approaches to Engage People Who Inject Drugs Into Care for Hepatitis C Virus Infection. Addict Disord Their Treat. 2017;16:S1–23. 28701904 10.1097/ADT.0000000000000104 PMC5491232

[27] Suffoletto B, Yanta J, Kurtz R, Cochran G, Douaihy A, Chung T. Acceptability of an Opioid Relapse Prevention Text-message Intervention for Emergency Department Patients. J Addict Med. 2017;11:475–82. 28858888 10.1097/ADM.0000000000000351 PMC5659903

[28] Tofighi B, Grossman E, Bereket S, Lee JD. Text message content preferences to improve buprenorphine maintenance treatment in primary care. J Addict Dis. 2016;35:92–100. 26670868 10.1080/10550887.2015.1127716

[29] Ashford RD, Lynch K, Curtis B. Technology and Social Media Use Among Patients Enrolled in Outpatient Addiction Treatment Programs: Cross-Sectional Survey Study. J Med Internet Res. 2018;20:e84. 29510968 10.2196/jmir.9172 PMC5861298

[30] Tofighi B, Grossman E, Sherman S, Nunes EV, Lee JD. Mobile Phone Messaging During Unobserved “Home” Induction to Buprenorphine. J Addict Med. 2016;10:309–13. 26933874 10.1097/ADM.0000000000000198

[31] Muench F, Weiss RA, Kuerbis A, Morgenstern J. Developing a theory driven text messaging intervention for addiction care with user driven content. Psychol Addict Behav. 2013;27:315–21. 22963375 10.1037/a0029963 PMC3531566

[32] Ranney ML, Choo EK, Wang Y, Baum A, Clark MA, Mello MJ. Emergency department patients’ preferences for technology-based behavioral interventions. Ann Emerg Med. 2012;60:218–27.e48. 22542311 10.1016/j.annemergmed.2012.02.026

[33] Fox S, Duggan M. Health online 2013. Health. 2013;2013:1–55.

[34] Better Help. 2019 Available from: https://www.betterhelp.com/faq/#96 Accessed 2019 Oct 2.

[35] Talkspace. 2019 Available from: https://www.talkspace.com/ Accessed 2019 Oct 2.

[36] Denecke K, Deng Y. Sentiment analysis in medical settings: New opportunities and challenges. Artif Intell Med. 2015;64:17–27. 25982909 10.1016/j.artmed.2015.03.006

[37] Hirschberg J, Manning CD. Advances in natural language processing. Science. 2015;349:261–6. 26185244 10.1126/science.aaa8685

[38] Yoon S, Parsons F, Sundquist K, Julian J, Schwartz JE, Burg MM, Comparison of Different Algorithms for Sentiment Analysis: Psychological Stress Notes. Stud Health Technol Inform. 2017;245:1292. 29295377 PMC5832438

[39] Yang H, Willis A, de Roeck A, Nuseibeh B. A hybrid model for automatic emotion recognition in suicide notes. Biomed Inform Insights. 2012;5:17–30. 22879757 10.4137/BII.S8948 PMC3409477

[40] Zou X, Yang J, Zhang J. Microblog sentiment analysis using social and topic context. PLoS One. 2018;13:e0191163. 29394258 10.1371/journal.pone.0191163 PMC5796698

[41] Jiang Y, McDonald JV, Wilson ME, Koziol J, Goldschmidt A, King E, Rhode Island Unintentional Drug Overdose Death Trends and Ranking—Office of the State Medical Examiners Database. Rhode Island Med J. 2018;101:33–6. 29393310

[42] Spirito A, Monti PM, Barnett NP, Colby SM, Sindelar H, Rohsenow DJ, A randomized clinical trial of a brief motivational intervention for alcohol-positive adolescents treated in an emergency department. J Pediatr. 2004;145:396–402. 15343198 10.1016/j.jpeds.2004.04.057

[43] Cunningham RM, Chermack ST, Zimmerman MA, Shope JT, Bingham CR, Blow FC, Brief motivational interviewing intervention for peer violence and alcohol use in teens: one-year follow-up. Pediatrics. 2012;129:1083–90. 22614776 10.1542/peds.2011-3419 PMC4074654

[44] Depp CA, Mausbach B, Granholm E, Cardenas V, Ben-Zeev D, Patterson TL, Mobile interventions for severe mental illness: design and preliminary data from three approaches. J Nerv Ment Dis. 2010;198:715–21. 20921861 10.1097/NMD.0b013e3181f49ea3 PMC3215591

[45] Suffoletto B, Callaway C, Kristan J, Kraemer K, Clark DB. Text-message-based drinking assessments and brief interventions for young adults discharged from the emergency department. Alcohol Clin Exp Res. 2012;36:552–60. 22168137 10.1111/j.1530-0277.2011.01646.x

[46] Whittaker R, Merry S, Stasiak K, McDowell H, Doherty I, Shepherd M, MEMO—A mobile phone depression prevention intervention for adolescents: development process and postprogram findings on acceptability from a randomized controlled trial. J Med Internet Res. 2012;14:e13. 22278284 10.2196/jmir.1857 PMC3846345

[47] Kalchbrenner N, Grefenstette E, Blunsom P. A Convolutional Neural Network for Modelling Sentences. Available from: http://arxiv.org/abs/1404.2188 Accessed 2019 Nov 27.

[48] Howard J, Ruder S. Universal Language Model Fine-tuning for Text Classification. Available from http://search.ebscohost.com.revproxy.brown.edu/login.aspx?direct=true&db=edsarx&AN=edsarx.1801.06146&site=eds-live&scope=site Accessed 2019 Nov 27.

[49] Sun Y, Wang S, Li Y, Feng S, Tian H, Wu H, ERNIE 2.0: A Continual Pre-training Framework for Language Understanding. Available from: http://arxiv.org/abs/1907.12412. Accessed 2019 Nov 27.

[50] Harris PA, Taylor R, Thielke R, Payne J, Gonzalez N, Conde JG. Research electronic data capture (REDCap)--a metadata-driven methodology and workflow process for providing translational research informatics support. J Biomed Inform. 2009;42:377–81. 18929686 10.1016/j.jbi.2008.08.010 PMC2700030

[51] Biedrzycki OJ, Bevan D, Lucas S. Fatal overdose due to prescription fentanyl patches in a patient with sickle cell/beta-thalassemia and acute chest syndrome: A case report and review of the literature. Am J Forensic Med Pathol. 2009;30:188–90. 19465816 10.1097/PAF.0b013e318187de71

[52] System Usability Scale. 1986 Available from: http://www.usability.gov/how-to-and-tools/resources/templates/system-usability-scale-sus.html Accessed 2015 Oct 17.

[53] Bangor A, Kortum PT, Miller JT. An empirical evaluation of the System Usability Scale. Int J Human-Computer Interaction. 2008;24:574–94.

[54] Lewis JR, Sauro J. The factor structure of the System Usability Scale In: Proceedings of the 1st International Conference on Human Centered Design: Held as Part of HCI International San Diego (CA, US): Springer-Verlag; 2009 p. 94–103.

[55] Attkisson CC, Zwick R. The Client Satisfaction Questionnaire: Psychometric properties and correlations with service utilization and psychotherapy outcome. Eval Program Plann. 1982;5:233–7. 10259963 10.1016/0149-7189(82)90074-x

[56] LeBlanc DM, McGinn MA, Itoga CA, Edwards S. The affective dimension of pain as a risk factor for drug and alcohol addiction. Alcohol. 2015;49:803–9. 26008713 10.1016/j.alcohol.2015.04.005 PMC4628900

[57] Wilson M, Gogulski HY, Cuttler C, Bigand TL, Oluwoye O, Barbosa-Leiker C, Cannabis use moderates the relationship between pain and negative affect in adults with opioid use disorder. Addict Behav. 2018;77:225–31. 29078148 10.1016/j.addbeh.2017.10.012

[58] Guest G, MacQueen KM, Namey EE. Applied thematic analysis. Los Angeles (US): Sage; 2011.

[59] Braun V, Clarke V. Using thematic analysis in psychology. Qual Res Psychol. 2006;3:77–101.

[60] QSR International. NVivo Qualitative Data Analysis Program (Version 10). [Computer software] Melbourne (Australia): QSR International; 2012.

